# Feasibility, engagement, and preliminary clinical outcomes of a digital biodata-driven intervention for anxiety and depression

**DOI:** 10.3389/fdgth.2022.868970

**Published:** 2022-07-22

**Authors:** Charalampos Tsirmpas, Dimitrios Andrikopoulos, Panagiotis Fatouros, Georgios Eleftheriou, Joaquin A. Anguera, Konstantinos Kontoangelos, Charalabos Papageorgiou

**Affiliations:** ^1^Feel Therapeutics Inc., San Francisco, CA, United States; ^2^Departments of Neurology and Psychiatry, University of California, San Francisco, San Francisco, CA, United States; ^3^First Department of Psychiatry, Eginition Hospital, Medical School National and Kapodistrian University of Athens, Athens, Greece; ^4^Neurosciences and Precision Medicine Research Institute “Costas Stefanis”, University Mental Health, Athens, Greece

**Keywords:** data-driven therapeutics, major depressive disorder, generalized anxiety disorder, psychophysiological data, emotion detection

## Abstract

**Hypothesis:**

The main hypothesis is that a digital, biodata-driven, and personalized program would exhibit high user retention and engagement, followed by more effective management of their depressive and anxiety symptoms.

**Objective:**

This pilot study explores the feasibility, acceptability, engagement, and potential impact on depressive and anxiety and quality of life outcomes of the 16-week Feel Program. Additionally, it examines potential correlations between engagement and impact on mental health outcomes.

**Methods:**

This single-arm study included 48 adult participants with mild or moderate depressive or anxiety symptoms who joined the 16-week Feel Program, a remote biodata-driven mental health support program created by Feel Therapeutics. The program uses a combination of evidence-based approaches and psychophysiological data. Candidates completed an online demographics and eligibility survey before enrolment. Depressive and anxiety symptoms were measured using the Patient Health Questionnaire and Generalized Anxiety Disorder Scale, respectively. The Satisfaction with Life Scale and the Life Satisfaction Questionnaire were used to assess quality of life. User feedback surveys were employed to evaluate user experience and acceptability.

**Results:**

In total, 31 participants completed the program with an overall retention rate of 65%. Completed participants spent 60 min in the app, completed 13 Mental Health Actions, including 5 Mental Health Exercises and 4.9 emotion logs on a weekly basis. On average, 96% of the completed participants were active and 76.8% of them were engaged with the sensor during the week. Sixty five percent of participants reported very or extremely high satisfaction, while 4 out of 5 were very likely to recommend the program to someone. Additionally, 93.5% of participants presented a decrease in at least one of the depressive or anxiety symptoms, with 51.6 and 45% of participants showing clinically significant improvement, respectively. Finally, our findings suggest increased symptom improvement for participants with higher engagement throughout the program.

**Conclusions:**

The findings suggest that the Feel Program may be feasible, acceptable, and valuable for adults with mild or moderate depressive and/or anxiety symptoms. However, controlled trials with bigger sample size, inclusion of a control group, and more diverse participant profiles are required in order to provide further evidence of clinical efficacy.

## 1. Introduction

Major depressive disorder (MDD) and generalized anxiety disorders (GAD) are very common but serious mental disorders that can lead to considerable deterioration in overall health and daily functioning levels. MDD and GAD are typically manifested by adverse effects on a person's thoughts, behaviors, motivation, feelings, and sense of well-being, as well as a major disruption of a person's day-to-day life. According to the Anxiety and Depression Association of America (ADAA) (1), 18.1% of the adult population in the US is affected by anxiety disorders every year, and approximately 7% shows symptoms of MDD on a yearly basis, with MDD being the leading cause of disability among people aged 15–45. Similarly for the European Union (EU), the Organisation for Economic Co-operation and Development (OECD) (2) estimates that anxiety disorders affected an estimated 5.4% of the population in 2016, which equates to approximately 25 million people, while a recent study (3) showed that more than 6% of the EU population suffers from depression (data collected during the period 2013−2015). The World Health Organization (WHO) estimates that 1 in 4 people in Europe are affected by depression or anxiety each year (4). Given the high prevalence of MDD and GAD worldwide, efforts to quantify and assess their impact on a person's health and functioning leverage the Years Lived with Disability (YLD) metric (5). Using this metric, the WHO reports (6) that depressive disorders rank as the most prevalent contributor to non-fatal health loss, representing 7.5% of the total YLD worldwide, whilst also being one of the leading causes of suicide. At the same time, anxiety which is generally associated with a lower average level of disability on average (sixth largest contributor) accounts for 3.4% of the total YLD globally (6).

Apart from the direct implications on overall health and wellbeing of those affected, MDD and GAD also heavily impact the global labor market. The OECD reports (7) a 15–30% decrease in the likelihood of employment and a twice as high unemployment rate for people with mental health disorders, such as anxiety and depression. Furthermore, compared to the mentally healthy, people with mental health issues are more likely to report job strain, and are on average 25% less satisfied with their jobs, with the majority of them earning less than the overall median income as well (4, 8). Moreover, an increase in chronic absences due to sickness can be expected due to the increased burden in both their personal and professional lives. Interestingly, in the EU (9), up to half of the total sick leaves regard depression or anxiety. Consequently, all these factors combined can lead to a significant reduction in work productivity. According to a study in several OECD countries (10), it is estimated that compared to the healthy workforce, employees with mental health disorders performed three times worse. It is evident therefore that MDD and GAD may add a heavy burden to the global budget as a result of the reduced labor market participation stemming from lower employment rates and reduced productivity owed to reduced job satisfaction and unavoidable sick leaves. Combined with an increase in spending for the associated social benefits, the total indirect costs of MDD and GAD can sum up to $1 trillion annually, worldwide (9).

As can be understood, diagnosing, supporting, managing, and treating MDD and GAD is of utmost importance. Numerous reports (11–14) suggest an increasing trend in the prevalence of MDD and GAD which is further accelerated by the recent COVID-19 pandemic (15–17). Consequently, an increasing number of people are expected to develop symptoms of depression and anxiety in the following years. Although there exist tools and treatments, more than half of people suffering from MDD or GAD face barriers that prevent them from accessing mental health care resources (18–20). Among these, lack of access and utilization of mental health services is prevalent particularly in developing countries (21–23), where policies, health services, and research regarding mental health are ill-represented in the countries' budget with respect to the size of the problem. This can lead to a threefold reduction in the probability of obtaining mental health care compared to the situation in developed countries (24, 25). Nevertheless, even in the case of more advanced countries, there exist a number of reasons that hinder access to mental health care. An indicative example regards that of the U.S. where a recent study (19) reported that almost half of the participants could not afford the cost of treatments, while approximately 17% cited reasons related to the lack of awareness of any services for reaching out. On a more individual-oriented level, more than 30% of participants raised concerns regarding social stigma, adverse effects on professional life, or unavailability for in-person treatment sessions.

Currently, traditional approaches for the management and treatment of depression and anxiety involve pharmacological treatment as well as psychotherapy sessions offered in the form of Cognitive Behavioral Therapy (CBT) or Interpersonal Therapy (IPT). Usually, for the more severe and chronic cases, a combination of both therapy and medication is followed (26). The efficacy of both approaches has been extensively investigated and has shown to be consistently high across many studies (27–29). Nevertheless, there exist additional factors that determine whether a specific approach proves beneficial for patients in the long run. The poorly established doctor-patient relationships due to lack of knowledge and insufficient training (27), the inability of patients to consistently follow psychotherapy sessions in the long run (19), along with the additional cost of medication and the adverse side effects that sometimes emerge (30), yield a risk of non-adherence and discontinuity of treatment (31, 32) which can reach values close to 60% for patients with depression or anxiety (30, 33). At the same time, subjective and patient-related factors (27) such as the severity of symptoms, comorbidities, and cultural beliefs, as well as limited response to medication due to underlying pathological conditions (27, 34) might hinder the overall efficacy of a treatment protocol, leaving an estimated 30%-40% of patients (27, 34) with minor or no symptom improvement.

Toward alleviating accessibility barriers and inequalities in mental health care, as well as potentially addressing the factors contributing to non-adherence and non-response to management and treatment protocols, digital mental health support tools have spurred during the last years and especially during the COVID-19 pandemic period (35, 36). These solutions revolve around therapeutic approaches and positive behavioral change, which can also work complementary to long-established traditional methods. Contrary to the latter, however, digital mental health offerings translate into remote and on-demand, personalized, inexpensive approaches for the treatment and management of mental health disorders. In this way, broader access to mental health care resources is achieved. The impact of such solutions has been extensively studied in the literature (37–41), with the results suggesting that an equal or greater effectiveness compared to traditional approaches can be achieved. Currently, there exist several products^1^ that primarily offer remote therapy sessions which can also be complemented by tutorials, educational/training material, and exercises adapted to the specific clinical case.

These years, we are experiencing an evolution of management and treatment solutions for various physical health disorders toward precise medicine expressed by quantifiable data-driven schemes and continuous monitoring approaches. This established paradigm guides us to a potentially promising alternative for mental health support. Such approaches have been shown to significantly alter current practices and introduce considerable improvements in treatment adherence and effectiveness, with diabetes and cardiovascular diseases being two representative examples. For the former, continuous blood sugar monitoring devices along with insulin pumps are combined in a smart hybrid device that automatically regulates the delivery of insulin based on real-time readings of blood sugar levels. Similarly, for cardiovascular diseases, the pacemakers act by sending electrical signals to increase the heartbeats when their sensors pick up bradycardia conditions. In both cases, it is the introduction of objective data, as reflected by physiological measurements (i.e., glucose and heart electrical activity, respectively) that has enabled real-time, inexpensive, and unobtrusive interventions. These are usually integrated into closed-loop telehealth solutions which show increased engagement, effectiveness, and improvement of the quality of life of patients in comparison to conventional therapies (42–46). Therefore, using continuous measurement and data-driven interventions for diagnosis, management or treatment are the way forward for mental health. This introduction of continuous, passive, and objective data, in conditions such as depression and anxiety, is expected to increase engagement, and facilitate personalized treatment approaches, which are expected to be more effective.

With respect to tackling some of the factors leading to patient non-adherence and non-response to management and treatment protocols, digital mental health tools can be augmented by multimodal, digital data, *via* the utilization of mobile phones and wearable sensors. This kind of data varies in complexity, ranging from straightforwardly interpreted mobile app-based data such as user interactions, app usage, and activity tracking *via* the embedded mobile phone sensors (i.e., GPS), to much more complex data that may require dedicated devices and/or advanced collection and processing techniques such as physiological signals, voice, text, etc. The expectation is that part, or all, of the acquired data, offer a degree of objectivity and ubiquitousness and as such, it enables a greater understanding and a deeper insight into the behavior of individuals, in the context of their respective mental health conditions. At the same time, by incorporating an adaptive UX/UI such as a gamified experience, it is claimed that long-term engagement can be realized, enhancing in this way the effectiveness of the solution (47–50) and reducing both any direct and indirect costs involved (51–54). Regardless of their obvious advantages, however, digital mental health solutions need to be largely scalable in order to achieve their full potential, while maintaining user engagement and treatment effectiveness.

Acknowledging the importance of increased accessibility to mental health care and the added value of multimodal and objective data, Feel Therapeutics^2^ has introduced the 16-week Feel Program (FP), which is a data-driven, digital mental health support program that uses a combination of emotion journaling, evidence-based cognitive behavioral therapy, mindfulness and positive psychology techniques augmented with the Feel Mental Health Biomarkers platform. Specifically, the FP consists of four components: (i) The Feel Emotion Sensor (FES), a wrist-worn device that provides continuous and unobtrusive monitoring of an individual's significant emotional changes. The FES continuously monitors the physiological signals of the user (i.e., Electrodermal Activity, Heart Rate Variability, and Skin Temperature) to detect changes in the activation of their Autonomic Nervous System and extract a series of other mental health metrics; (ii) The Feel mobile app, which utilizes data from the FES to provide near real-time emotion alerts and interventions, access to other mental health-related metrics and facilitate virtual sessions; (iii) Personalized weekly 15-min coaching sessions with the Feel Providers, augmented by the weekly extracted mental health metrics such as emotion-related, and self-reported data, etc.; (iv) Mental health resource center that compiles tutorials, exercises, tips and advice focusing on the development of mental health coping skills.

In order to explore and evaluate the feasibility, acceptance, and potential efficacy of the FP, a real-world data (RWD) feasibility study has been designed and conducted. In particular, focusing on mild or moderate MDD and/or GAD, Feel Therapeutics has designed and conducted a Proof-of-Concept (PoC), single-arm pilot study, aiming to (i) explore the ecological validity of the Feel emotion detection technology, (ii) validate the engagement with and (iii) evaluate the preliminary efficacy of the FP. The main hypothesis is that a remote, data-driven, and personalized program would exhibit increased levels of user retention and engagement during the 16-weeks, followed by a reduction of their depression and anxiety symptoms that would be captured by a respective decrease in associated clinical measures. In this work, we attempt to validate this hypothesis by presenting the main results of this PoC study, including (i) adoption/conversion rates; (ii) engagement levels with the program, along with the respective drop-out rates; (iii) the impact of the program on health-related quality of life; (iv) preliminary efficacy of the program; (v) validation of the emotion detection capabilities of the Feel technology measured at an in-the-wild environment.

The rest of this work is organized as follows: in Section 2, a detailed description of the materials and methods used for the PoC study is provided. Furthermore, in Section 3, we present the results of the study focusing on the feasibility and acceptability of the program (Section 3.1), the ecological validation of the Feel Emotion Detection technology (Section 3.2), the participant retention and engagement in the program (Section 3.3), as well as a preliminary assessment of the program's impact on mental health symptoms (Section 3.4), quality of life measures (Section 3.5) and participant self-assessment metrics (Section 3.6). Then, in Section 4 we continue with a thorough discussion, presenting an interpretation of the results toward supporting our hypothesis (Section 4.1), along with a few further observations (Section 4.2. Finally, the study limitations are outlined in Section 4.3 and this work is concluded in Section 4.4.

## 2. Materials and methods

### 2.1. Participant recruitment

The company developed a dedicated web page that included information about the Feel Program for depression and anxiety for participant recruitment.The target audience for the study was general public, while the study recruitment process was advertised *via*: (i) candidate referrals from the undergraduate student mental health support unit of the National and Kapodistrian University of Athens; (ii) social media ads (e.g., Facebook, Instagram, etc.) and (iii) word of mouth.

The study's inclusion and exclusion criteria were assessed based on self-reported candidate responses to the eligibility questionnaire, while they were also verified by the Feel Providers during the first introductory session. The main inclusion criteria were: (i) mild to moderate MDD (4 < PHQ-9 < 15) and/or GAD (4 < GAD-7 < 15); (ii) age ≥18 years old; and (iii) smartphone users/owners. On the other hand, the main exclusion criteria were: (i) severe MDD and/or GAD; (ii) personality disorders; (iii) psychotic disorders; (iv) bipolar disorder; (v) eating disorders; (vi) suicidal or self-harm thoughts; (vii) psychotropic medication; (viii) substance abuse.

### 2.2. Materials

#### 2.2.1. Demographic and Eligibility Questionnaire

This questionnaire was completed by candidates at the screening stage, in order to assess their eligibility and included demographic information (e.g., gender, age, location, etc.), presence of any of the exclusion criteria, along with wrist measurements, in order to determine the appropriate sensor size, in case of a positive eligibility assessment. The Demographic and Eligibility Questionnaire was embedded at the recruitment web page and completed at baseline.

#### 2.2.2. Patient Health Questionnaire 9-item (PHQ-9)

The PHQ-9 is a self-administered questionnaire for assessing the severity of depressive symptoms (55). The questionnaire is composed of 9 items, each one scoring the frequency of occurrence of the 9 DSM-IV criteria on a scale from 0 (not at all) to 3 (nearly every day). The total score is the sum of the scores of the individual items. The threshold scores for classifying the severity of the depressive symptoms as mild, moderate, moderately severe, and severe depression are 5, 10, 15, and 20, respectively. The PHQ-9 was completed at baseline (embedded in the recruitment web page) and at weeks 8 (mid-program) and 16 (end-of-program) in the Feel app.

#### 2.2.3. Generalized Anxiety Disorder-7 (GAD-7)

The GAD-7 is a self-administered questionnaire that serves as a brief clinical measure for assessing the severity of GAD (56). The questionnaire is composed of 7 items regarding DSM-IV criteria, each one scoring the frequency of occurrence of symptoms on a scale from 0 (not at all) to 3 (nearly every day). Based on the total score, which is the sum of the individual scores from the 7 items, the severity of the anxiety symptoms is assessed. More specifically, for the classification of the symptoms as mild, moderate, or severe anxiety, threshold scores of 5, 10, and 15 have been used, respectively. The GAD-7 was completed at baseline (embedded in the recruitment web page) and at weeks 8 (mid-program) and 16 (end-of-program) in the Feel app.

#### 2.2.4. Life Satisfaction Questionnaire (LISAT-11)

The LISAT-11 questionnaire is a self-administered tool for measuring Life Satisfaction (57). It is comprised of 11 items; 1 global item evaluating life as a whole, and 10 domain-specific items including vocational situation, financial situation, leisure, contact friends, sexual life, activities of daily living, family life, partnership relationship, physical health, and psychological health. Each item is scored on a range from 1 to 6, with the total score being the mean of the individual scores. Higher scores indicate a greater level of perceived life satisfaction. The LISAT-11 was completed at baseline (embedded in the recruitment web page) and at weeks 8 (mid-program) and 16 (end-of-program) in the Feel app.

#### 2.2.5. Satisfaction With Life Scale (SWLS)

The SWLS is a self-administered subjective well-being questionnaire that measures global life satisfaction (58). It consists of 5 items, each one scored from 1 (strongly disagree) to 7 (strongly agree) with the cutoff scoring values being 10, 15, 20, 21, 26, and 31, for life satisfaction levels ranging from extremely dissatisfied to extremely satisfied. The SWLS was completed at baseline (embedded in the recruitment web page) and at weeks 8 (mid-program) and 16 (end-of-program) in the Feel app.

#### 2.2.6. Self-assessment questionnaire

The self-assessment questionnaire is a custom tool, aiming to capture the participants' perception regarding their accomplishments and progress throughout the 16 weeks of the program. Indicative questions of this survey are: “My concerns that brought me to the program have improved as a result of the services provided” and “I learned to think more clearly/accurately to reduce distressing emotions or behaviors,” among others. A 5-point Likert-type scale was used with participant responses ranging from 1 (strongly disagree) to 5 (strongly agree). The self-assessment questionnaire was completed at week 16 (end-of-program) in the Feel app.

#### 2.2.7. Mobile app interaction metrics

A wide range of mobile-app-related metrics was collected during the 16 weeks of the program, including participants' responses to emotion notifications, number of times each component of the FP was accessed, exercises completed, time spent in the Feel app, number of weekly sessions with the Feel provider attended, etc.

#### 2.2.8. User feedback survey

After the completion of the program (i.e., at 16 weeks), the participants were asked to complete a feedback survey (administered in the Feel app), in order to identify and measure various aspects of their experience throughout the program. The first part of the survey addressed the overall level of satisfaction with the program. Then, a group of questions helped to assess the ease of use of the different program components (e.g., FES, Feel app, etc.) and the responsiveness of Customer Support. Next, questions on the importance and value of each program component followed. Finally, the participants were given the option to provide open-ended comments on aspects and features of the FP they particularly liked or considered useful, as well as recommendations for improvements. A 5-point Likert-type scale was used with participant responses ranging from 1 (not at all) to 5 (extremely).

### 2.3. Methods

#### 2.3.1. Participant screening and onboarding flow

The study design was single-arm and was conducted in accordance with the Declaration of Helsinki and was approved by the Ethics Committee of the National and Kapodistrian University of Athens, 1^st^ Department of Psychiatry, Eginition Hospital. The study took place between January 2020 and October 2020. Firstly, individuals interested in participating in the study should complete an online demographic and eligibility questionnaire, as well as the PHQ-9 and GAD-7. The applicants' eligibility was evaluated considering their self-reported survey responses, along with the study's inclusion and exclusion criteria (see Section 2.2). Candidates who fulfilled any of the exclusion criteria were immediately disqualified and received proper communication accordingly. Prior to the start of the program, eligible participants were provided a comprehensive description of the Program and study's scope and components, and scheduled an orientation session with a member of our customer success team that would introduce and onboard them on the program components.

**Figure 1:**
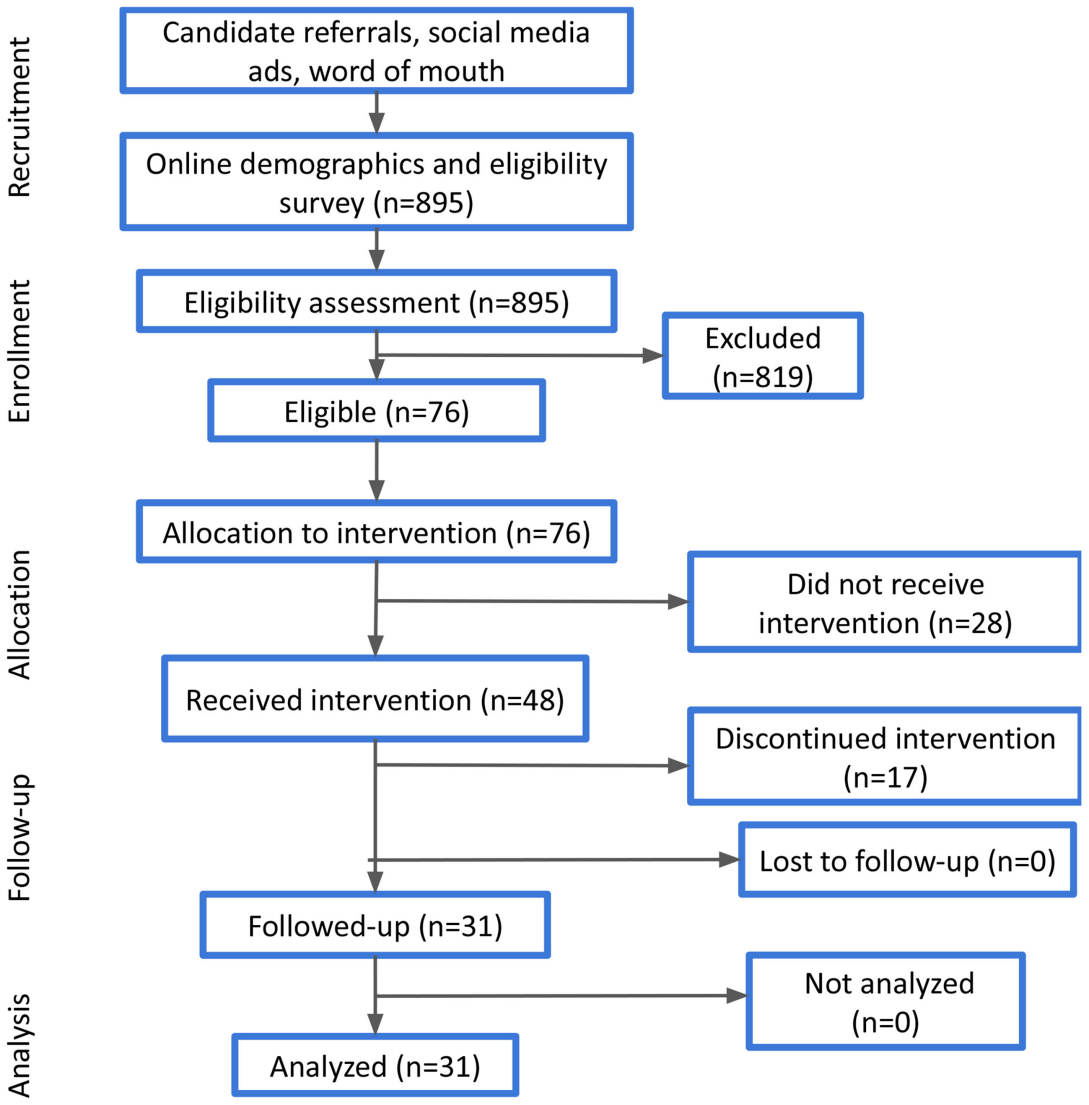
Overview of the participant screening and onboarding flow.

Participants that have accepted the invitation to join the study were provided with a Feel Emotion Sensor, downloaded the Feel app, and attended the orientation session, where they were familiarized with the basic components of the program and the key functionalities of the sensor. As a next step, they had to register to the Feel app and complete the onboarding quests that aim to walk them through the different parts of the program. Among these, participants could check the providers' availability, select their preferred one and book their 16 weekly sessions. All participants who completed these steps finally joined the study. Their progress was monitored at the middle (i.e., after the 8^th^ session) and at the end of the study (i.e., after the 16^th^ session). Participant demographics (e.g., age, gender, etc.) that were acquired at the completion of the online demographic survey were also verified during the orientation session. A high-level presentation of the experimental process is illustrated in Figure 1.

#### 2.3.2. Feel Program

**Figure 2:**
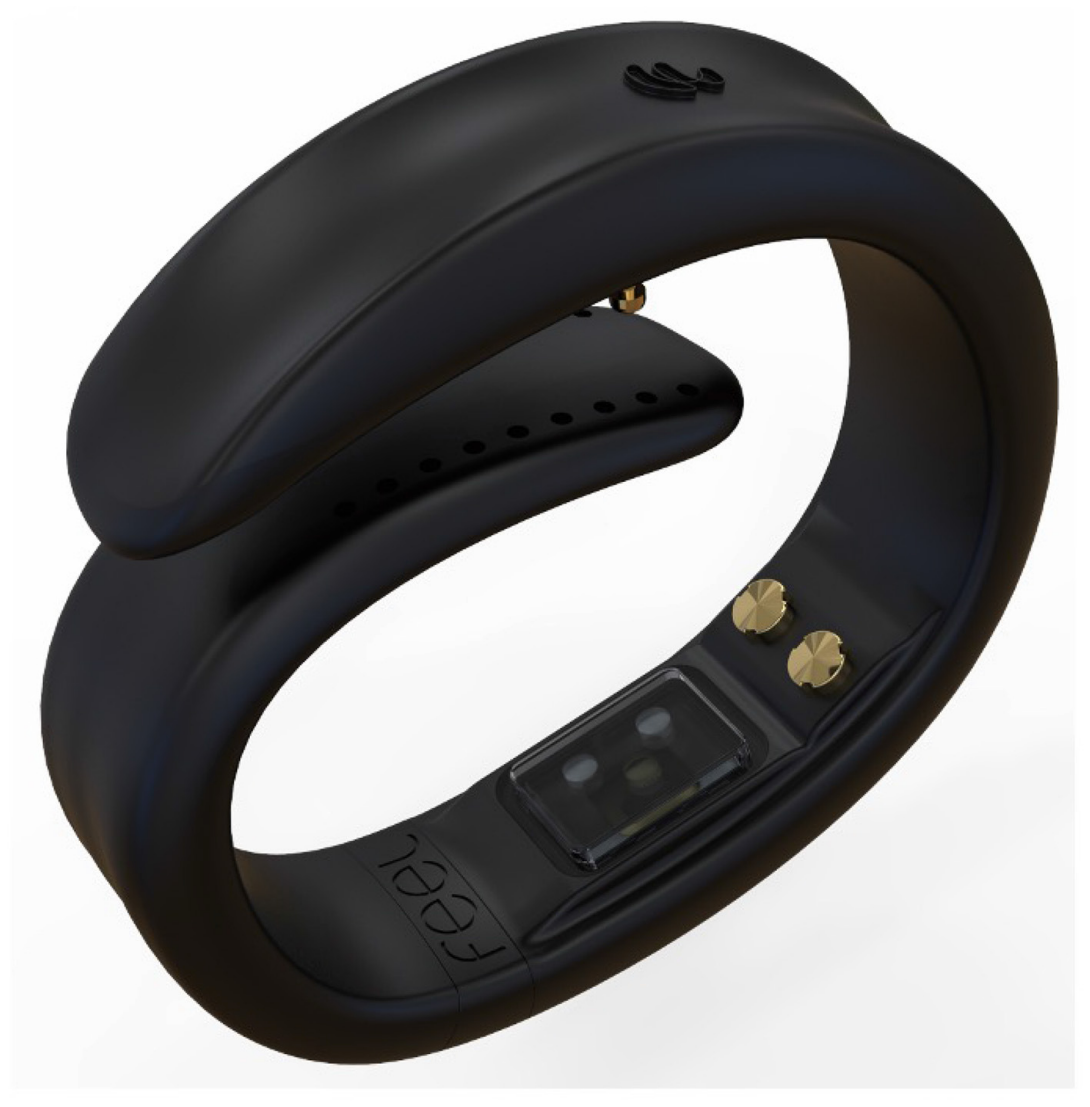
The Feel Emotion Sensor (FES).

The Feel Program (FP) is a fully-remote mental health support program created by Feel Therapeutics, in an effort to bring objective data and precise therapeutics in the management and treatment of mental health conditions and address the ever-increasing need of people to enhance emotional awareness and self-regulating skills. The program uses a combination of emotion journaling and evidence-based approaches, such as cognitive behavioral therapy (CBT), mindfulness and Positive Psychology (PP) techniques. Furthermore, it is augmented by physiological data that reflect the activation of the individual's Autonomic Nervous System and capture mental health and emotion-related information. The program is available as a patient support system in the USA and Europe and is available for download *via* the App Store (iOS) or Play Store (Android). For the present study, the FP focuses on people suffering from depression and/or anxiety and expands over 16 weeks and consists of the following components:

- **Feel Emotion Sensor**: The Feel Emotion Sensor (FES) is a wrist-worn electronic device designed and manufactured by Feel Therapeutics (Figure 2). The device consists of three main sensors for ubiquitous and unobtrusive monitoring of physiological signals: (i) a custom proprietary electrodermal activity (EDA) sensor measuring changes in skin conductance, (ii) an off-the-shelf photoplethysmogram (PPG) sensor for measuring Heart Rate and Heart Rate Variability (HRV) and (iii) an off-the-shelf temperature sensor for measuring skin temperature (ST). In addition, the FES contains a 9-axis Inertial Measurement Unit (IMU) serving as an accelerometer, gyroscope, and magnetometer. It also contains standard equipment adhering to the Bluetooth (BLE) protocol for pairing with a mobile device using the Feel App. Finally, the FES has obtained the following certifications: CE-RED (EN55032, EN61000, EN55035, EN301489, EN62368, EN300328, EN62479), IEC62133, WEEE, RoHs 2.0, FCC ID, FCC sDoc, CEC, US CA Prop 65, BQB/Bluetooth SIG, UN38.3 and MSDS.
- **Feel Mobile App**: The Feel Mobile App is a mobile application available in Android and iOS. It connects to the FES, collects data, and transfers it from the FES to the Feel cloud-based processing infrastructure. The Feel Mobile App helps the participants to onboard the Feel Program, guiding them on how to connect and use the FES, providing information on the program and the theory behind emotion journaling, and facilitating the scheduling of weekly sessions with their providers. Furthermore, the app responds in real-time to changes in the emotional state of the participants and helps them journal the emotions they experience. Access to weekly educational material that explains the evidence-based practices used in the program is offered, as well as default and personalized exercises to practice the various concepts. Self-guided tools such as mood boosters that increase engagement and further help the participants to reach their goals are integrated into the app. Moreover, the clear and coherent program structure and the progress bar within the app ensure that the participants can effortlessly track their progress in the program anytime.
- **Feel Mental Health Biomarkers platform**: The Feel Mental Health Biomarkers platform focuses on the discovery, extraction, and validation of mental health-related biomarkers and metrics for various mental and physical (where comorbid mental health conditions emerge) health use cases. In the context of this study, the Feel Emotion Detection (FED) has been the main functionality that has been deployed. The FED is the backbone of the Feel Program and is based on affective computing technology principles, translating physiological signals (i.e., EDA, HRV, and ST) to emotional events. The platform brings together many different data processing and insights extraction components, including data curation, artifact detection, signal processing and denoising, dynamic segmentation, feature extraction, personalization, and decision models.The FED infrastructure was developed and extensively tested by Feel Therapeutics, is proprietary and protected by U.S. patent (59).
- **Personalized weekly sessions**: The weekly sessions are administered remotely *via* teleconference, by the Feel Providers and have a duration of 15 min (apart from the 1st introductory session that lasts 45 min), augmented by the data provided by the Feel Emotion Sensor and the Feel app. There are a total of 16 sessions with weekly educational material and exercises. The first 3 weeks of the program are foundational and build upon the participants' knowledge. The following 5 weeks focus on CBT, biopsychology and positive psychology and the final weeks build upon the Thoughts, Feelings, Behavior cycle with developing skills and resiliency. The Feel Provider utilizes the Feel Dashboard to access the data to identify themes, key words and behavior patterns to prepare for the session in order to connect-the-dots with the weekly material and exercises. During the session, the provider is able to target feedback and personalize interventions based on each participant's data. The provider also discusses the participant's next steps after graduation and how they can continue applying what they learned to their daily lives.
- **Mental health resource center**: This component is directly integrated into the Feel mobile app and compiles tutorials, exercises, and tips that focus on the challenges present when dealing with depression and anxiety. The information is designed to engage the participant on their journey and provide the scientific basis for the program interventions and tools. The exercises compliment the material and are customized by the provider based on the participant's data. The participant establishes motivation to engage in the program, set program goals and enhance the knowledge with theoretical frameworks and evidenced-based practices. The educational material and exercises assigned promote mind-body awareness and self-management to further improve the quality of life according to the goals of the participant. The goal is to help participants develop mental health coping skills. All of the material in the mental health resource center has been created by Feel Therapeutics utilizing evidence-based techniques, including CBT, Mindfulness, Biopsychology and Positive Psychology, and is available to the participants anytime *via* the Feel mobile app.

#### 2.3.3. Emotion journaling

The emotion journaling aspect constitutes one of the core components of the data-driven nature of the FP. Thus, an engaging and intuitive journaling user experience has been designed and implemented in the Feel app. This journaling process integrates all the information required both for personalized interventions and for empowering the providers with emotion-related insights and patterns that could be leveraged during the weekly sessions. Additionally, it is used for algorithmic validation purposes, as well as improvements and enhancements of the Feel Biomarkers platform. The emotion journaling process can be either triggered by an emotion notification received by the FES (i.e., FES-triggered) or be manually logged by the participant. For each emotional event detected by the platform, the participant receives a notification in the Feel app to register their response to the detected event. The participant is presented with two high-confidence answers, “accept” or “reject” and with two low-confidence ones, “skip” and “not sure.” In case of an accurate detection (i.e., accepted event), the participant is also asked to specify the perceived intensity of the emotional event, ranging from 1 to 10, in the next mandatory step. Furthermore, participants are encouraged to input the triggers, thoughts, behaviors, and physical sensations associated with the specific emotional event in free-form text or voice recording in the following non-mandatory steps. It should be noted that for the emotional events manually registered by the participants (i.e., participant has not received an emotion notification), the journaling procedure is exactly the same, except for the first step requiring the response to a detected event. The complete structure for the accurately detected events involving all user input (both mandatory and not) is referred to as an emotion journal, while the sole completion of the mandatory steps constitutes an emotion log. All emotion logs can be accessed *via* the Feel app anytime throughout the program.

#### 2.3.4. Mid- and end-of-program assessment

Upon the completion of the 8^th^ week of the program, participants were asked to complete a set of questionnaires assessing their depressive and anxiety symptoms, as well as their life satisfaction levels, *via* the Feel app. Finally, at the end of the program participants responded to the same questionnaires, along with the self-assessment and the user feedback survey. No monetary incentive was provided to the participants.

#### 2.3.5. Statistical analysis

Regarding the FP feasibility assessment, the overall onboarding process is summarized and presented, followed by the participant responses to the eligibility survey. Considering that this is a PoC single-arm study that serves as a preliminary evaluation of the participant engagement with the intervention, followed by a preliminary assessment of the efficacy of the intervention, we have followed a per-protocol analysis approach. As stated by the FDA “the use of the per protocol set may maximize the opportunity for a new treatment to show additional efficacy in the analysis, and most closely reflects the scientific model underlying the protocol” (60). Therefore, adopting such an approach provides an opportunity to assess the preliminary effect of receiving the assigned intervention (61), which is the primary focus of this study. In the context, when evaluating engagement or preliminary efficacy aspects, only participants who have completed the study have been considered (i.e., 31 participants). We define a completed participant in our analysis as a participant who has not explicitly requested to withdraw from the study, regardless of their engagement level with the various study components (e.g., assessment surveys, sensor, weekly sessions, etc.).

For the evaluation of engagement metrics, the average values and percentages of participants engaging with the different program components are presented to evaluate engagement aspects. For such metrics, we present the aggregate values over a specified period of time (e.g., day, week, etc.), averaged over the number of participants. As previously discussed, for this analysis only the participants that completed the study have been considered. Finally, aggregate values of the participant responses to the user feedback survey have been employed to assess program acceptability.

With respect to the preliminary assessment of the intervention effect on depressive and anxiety symptoms, as well as on the participants' quality of life and life satisfaction levels, average values at baseline, mid-program and end-of-program have been used. The statistical significance of the results has been validated using the Wilcoxon signed-rank test and matched pairs rank-biserial correlation (*r*) for the effect sizes. Minimal clinically important differences (MCID) are defined as at least five points for the PHQ-9 scores (62) and at least four points for the GAD-7 scores (63). According to the per-protocol analysis methodology followed, only participants who have completed the program have been included in the analysis. The missing values accounted for 6.5% of the total assessment survey values that were used in the analysis and were imputed by utilizing the multivariate feature imputation available in the open source scikit-learn python package (64).

Finally, the different types of data collected from the participants during the eligibility, onboarding and assessment process, their physiological data, as well as data from the Feel app are stored and processed in our secure cloud-based infrastructure in Europe. For privacy reasons and in order to adhere to GDPR regulations, all data has been pseudonymized before any processing and insights extraction.

## 3. Results

### 3.1. Recruitment, feasibility, and acceptability

During the recruitment and onboarding process, 895 candidates answered the online demographic and eligibility survey, out of which 76 were eligible and considered to receive the intervention, while finally 48 participants actually joined the program. Candidate demographics, as well as responses to the various eligibility questions are presented in Table 1, along with the respective percentages. It should be noted that the overall eligibility ratio is not derived as the combination of the different percentages of the non-eligible responses, as many candidates may have had more than one condition (e.g., severe MDD and eating disorder, psychotic disorder and psychotropic medication). Thus, the overall eligibility ratio was 8.5%.

**Table 1:** Candidate responses to the demographic and eligibility questionnaire.

**Candidate responses**	
**Age, *n* (%)**	
<18	17 (1.9%)
18 or older	878 (98.1%)
Gender, *n* (%)	
Female	682 (76.2%)
Male	213 (23.8%)
**Income level, *n* (%)**	
<5, 000	150 (16.8%)
5, 001−15, 000	243 (27.2%)
15, 001−25, 000	158 (17.6%)
25, 001−50, 000	134 (15%)
>50, 000	38 (4.2%)
No response	172 (19.2%)
**Education, *n* (%)**	
No schooling completed	23 (2.6%)
High school graduate	260 (29%)
Bachelor's degree	170 (19%)
Master's degree	132 (14.7%)
Professional degree	254 (28.4%)
Doctorate degree	14 (1.6%)
No response	42 (4.7%)
Exclusion disorders^*^, *n* (%)	
Yes	220 (24.6%)
No	675 (75.4%)
**Psychotropic medication, *n* (%)**	
Yes	324 (36.2%)
No	571 (63.8%)
**Suicidal or self-harm thoughts, *n* (%)**	
Yes	128 (14.3%)
No	767 (85.7%)
**Substance abuse, *n* (%)**	
Yes	63 (7%)
No	832 (93*%*)
**MDD severity, *n* (%)**	
Minimal	105 (11.7%)
Mild-Moderate	464 (51.8%)
Moderately Severe-Severe	278 (31.1%)
No response	48 (5.4%)
**GAD severity, *n* (%)**	
Minimal	123 (13.8%)
Mild-Moderate	517 (57.8%)
Severe	200 (22.3%)
No response	55 (6.1%)

* *Exclusion disorders were: personality disorders, psychotic disorders, bipolar disorder, eating disorders*.

Out of the 48 participants who were invited to and joined the Program, all of them downloaded the Feel Mobile App, attended the orientation session, scheduled their weekly sessions with the Feel Provider and joined at least one session. Additionally, their mean age was 37.67 (*SD* = 10.11), with almost 70% of them being 18–40 years old, while their gender distribution was 62.5% females-37.5% males (Table 2). The average baseline PHQ-9 score was 9.1, where 50% of participants had mild depressive symptoms, 39.6% moderate and the rest minimal. Similarly, the average baseline GAD-7 score was 7.7, where 52.1% of participants had mild anxiety symptoms, 29.2% moderate and the remaining minimal. Moreover, 31 participants completed the Program, while 17 discontinued for various reasons, ranging from technical challenges when using the FES or the app to unwillingness and limited time availability to commit to the program.

**Table 2:** Participant demographic characteristics and baseline assessment scores.

**Participant characteristics**	
**Age, *n* (%)**	
18−30	13/48 (27.1%)
31−40	20/48 (41.7%)
41−50	9/48 (18.7%)
Older than 50	6/48 (12.5%)
**Gender, *n* (%)**	
Female	30/48 (62.5%)
Male	18/48 (37.5%)
**MDD symptom severity, *n* (%)**	
Minimal	5 (10.4%)
Mild	24 (50%)
Moderate	19 (39.6%)
**GAD symptom severity, *n* (%)**	
Minimal	9 (18.7%)
Mild	25 (52.1%)
Moderate	14 (29.2%)

Participant responses to the user feedback survey are presented in Table 3, with a 5-point Likert-type scale being used, where it becomes evident that the overall participant satisfaction levels are quite high with an average of 4 out of 5. More than 65% of participants are reporting at least very high satisfaction levels. Similarly, the average Net Promoter Score is also 4 out of 5, as it would be highly or extremely likely for close to 80% of participants to recommend the FP to someone they know. Regarding the usability of the different program components, 70% of participants found it easy to use the Feel Mobile app, while only 15% of participants seem to have had faced technical difficulties engaging with the FES. The level of responsiveness to questions or concerns about the FP (i.e., Customer Support) has received a remarkable 4.5 out 5 average rating with close to 90% of participants being very or extremely satisfied. Furthermore, regarding the importance of the different program components, the personalized data-driven sessions stand out with over 80% of participants perceiving them as very important, while 70% of participants identify the FES as a very important program component. Finally, participants identified 1) the emotion journaling flow, 2) the integration of their physiological data into the Program guideline and 3) the in-the moment interventions as the most important features of the program. On the other hand, the option to visualize the collected data (e.g., heart rate), the sensor ergonomics and battery life, along with the enhancement of the interactive material (e.g., weekly exercises) have emerged as the features that could be improved or added to the program.

**Table 3:** Participant responses to the user feedback survey.

**Survey questions**	**Participant responses**, *n* **(%)**
Participant satisfaction	
Overall, how satisfied are you with the Feel Program?	Extremely: 11 (36.7%) Very: 9 (30%) Neutral: 9 (30%) Slightly: 1 (3.3%) Not at all: 0 (-)
How likely are you to recommend the Feel Program to someone?	Extremely: 10 (33.3%) Very: 14 (46.7%) Neutral: 3 (10%) Slightly: 3 (10%) Not at all: 0 (-)
**Program components usability**	
How easy is it to navigate the Feel app?	Extremely: 9 (30%) Very: 12 (40%) Neutral: 3 (10%) Slightly: 5 (16.7%) Not at all: 1 (3.3%)
How easy is it to use the Feel emotion sensor?	Extremely: 6 (20%) Very: 7 (23.3%) Neutral: 12 (30%) Slightly: 3 (10%) Not at all: 2 (6.7%)
**Customer support**	
How responsive have we been to your questions or concerns about the Feel program?	Extremely: 20 (66.7%) Very: 6 (20%) Neutral: 4 (13.3%) Slightly: 0 (-) Not at all: 0 (-)
**Program components importance**	
Feel Emotion Sensor	Extremely: 14 (46.7%) Very: 7 (23.3%) Neutral: 6 (20%) Slightly: 1 (3.3%) Not at all: 2 (6.7&)
Feel Mobile App	Extremely: 14 (46.7%) Very: 7 (23.3%) Neutral: 3 (10%) Slightly: 6 (20%) Not at all: 0 (-)
Mental Health Resource Center	Extremely: 14 (46.7%) Very: 13 (43.3%) Neutral: 3 (10%) Slightly: 0 (-) Not at all: 0 (-)
Personalized Data-driven Sessions	Extremely: 22 (73.3%) Very: 3 (10%) Neutral: 5 (16.7%) Slightly: 0(-) Not at all: 0 (-)
Open-ended feedback^*^	
Features participants particularly liked	Emotion journaling flow: 15 (68.2%) Data integration: 12 (54.5%) In-the-moment interventions: 8 (36.4%)
Features to improve/add	Physiological data visualization: 8 (36.4%) Sensor ergonomics or battery life: 7 (31.8%) Enhance interactive material: 5 (22.7%)

* *Participants could select up to 3 features, so percentages do not add up to 100%*.

### 3.2. Ecological validity of the Feel Emotion Detection

As previously discussed, among the primary aims of this study was to validate in-the-wild the performance of the FED that focuses on the identification of the significant emotional moments participants have been experiencing. In this context, an analysis of the FES-triggered emotion logs follows, considering the participants' responses (positive or negative) on the notifications sent, as well as the perceived intensity input by the participants during the emotion logging flow. Overall, the average precision levels, that reflect the ratio of total accepted notifications of the study to the sum of the accepted and rejected, were 87%. On an individual level, the mean precision among the participants was 88% with a standard deviation of 0.2. Moreover, when weighing each participant's precision with the total number of notifications, the obtained (weighted) average precision was 86%. Furthermore, it should be noted that 75% of the participants had a precision of at least 85% (25^th^ percentile). The combination of the above supports the capability of FED to correctly identify the participants' emotional events in the wild.

**Figure 3:**
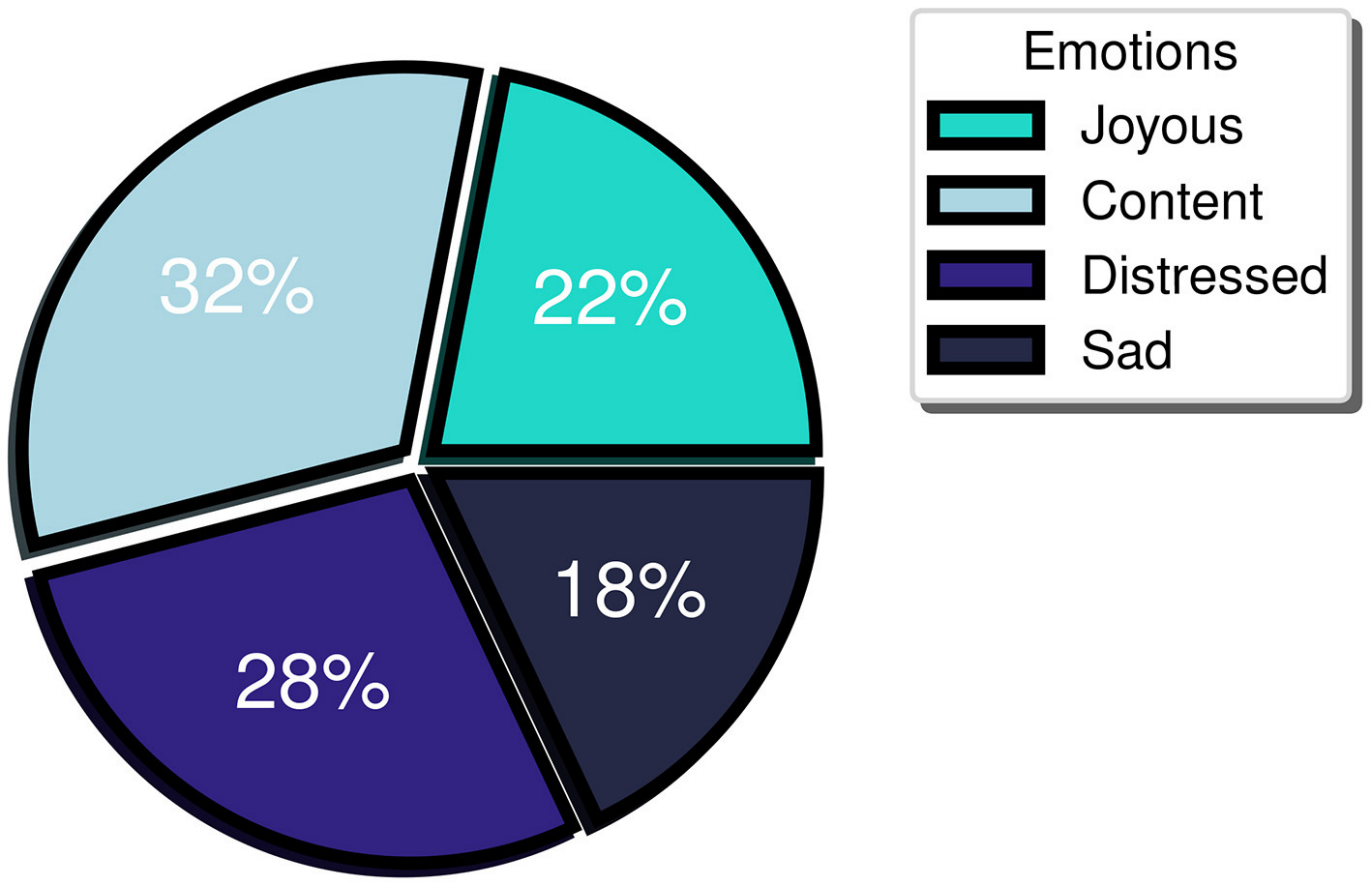
Distribution of emotions logged by type of emotion.

Furthermore, a balanced distribution between positive and negative emotional events was observed (54 vs. 46%, respectively) indicating minimum bias toward the detection of emotions of a particular valence. More specifically, when referring to the positive valence emotions, study participants logged 32% “content” and 22% “joyous” emotions, while for the negative valence ones, they logged 18% “sad” and 28% “distressed” (Figure 3). Meanwhile, regarding the participant-perceived emotion intensity, the mean intensity level logged was 6. Considering that participants may label their emotion intensity at a scale of 1 to 10, an intensity threshold at the midpoint of the scale (i.e., 5) has been selected, with emotional events rated 6 or higher perceived as high intensity, while the ones logged with 1 to 5 as low intensity. During the study, high intensity emotion logs constituted more than 60% of the total FES-triggered emotion logs. Furthermore, the mean intensity level of the accurately detected events for the majority (> 70%) of the participants was at least 6. The last two observations support that the FES captures the higher intensity (and perhaps more meaningful events, at least as experienced by each participant) with a great degree of uniformity across multiple individuals.

### 3.3. Participant retention and engagement in the FP

**Figure 4:**
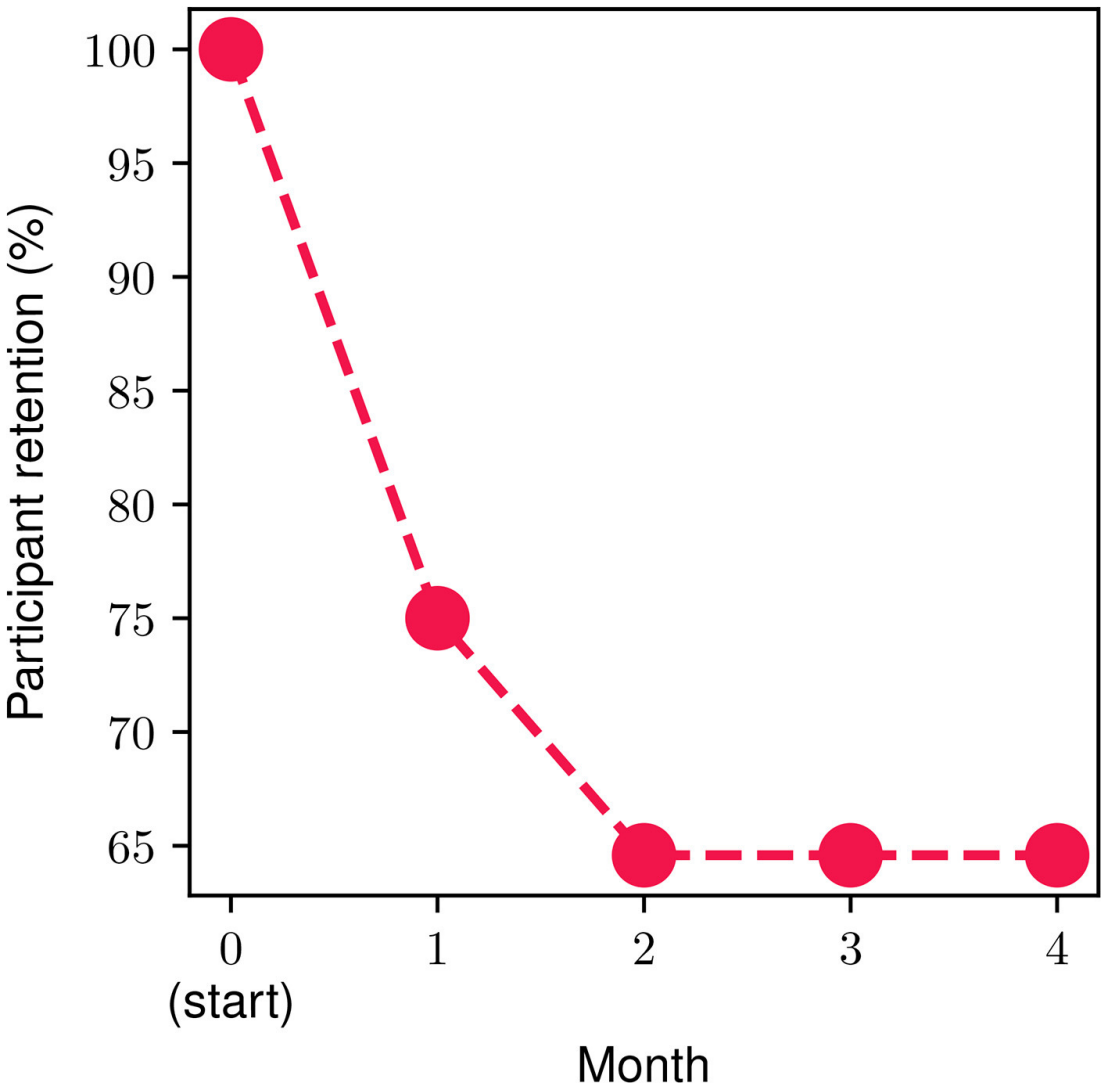
Participant monthly retention during the program.

In order to measure and extract meaningful insights associated with the participant retention levels in the FP, we divide the FP into four parts (modules), each one consisting of four personalized weekly sessions spread across a time period of 1 month. The monthly retention rate is illustrated in Figure 4. About 25% of the onboarded participants discontinued the program during the first month, while approximately 15% of the ones who went through the 1st month discontinued during the second one. Then, 100% of participants who completed the first 8 weekly sessions (i.e., the second monthly module) continued toward full completion of the FP. In other words, zero dropouts were observed after the second month. The overall retention throughout the study was 65%. The metrics presented in the remainder of this section refer to the participants that completed the study (*n* = 31, 65% of the total participants).

Regarding participant engagement, we gauge it by metrics that capture all the different aspects of the FP. We define as “active,” a completed participant that shows engagement with one or more of the study components (e.g., FES, Mental Health resources, etc.). With respect to the overall activity in the FP during the study, it was observed that participants engaged with any component of the FP on average for 3.1 days per week, with an average of 96% of them being active on a weekly basis. The participants spent close to 60 min per week (*SD* = 10.3) in the Feel Mobile app and accessed it on average for 16.5 times throughout the week which translates to roughly 2.5 times per day. Moreover, regarding the weekly data-driven sessions, we observed that the session compliance ratio was 96.9%, while on average, the participants completed 5 mental health exercises/educational material provided *via* the mental health resource center of the mobile app per week. Aiming to capture the overall participant engagement and their progress/efforts toward improving their mental health, an aggregate engagement metric, the weekly Mental Health Actions (MHA), is introduced. This metric includes the number of emotion logs, completed mental health exercises/educational material, sessions with the Provider attended, as well as whether the participant has engaged with the sensor or not and is calculated on a weekly basis. During this study, participants' MHA reached an average of 13 actions per week.

**Figure 5:**
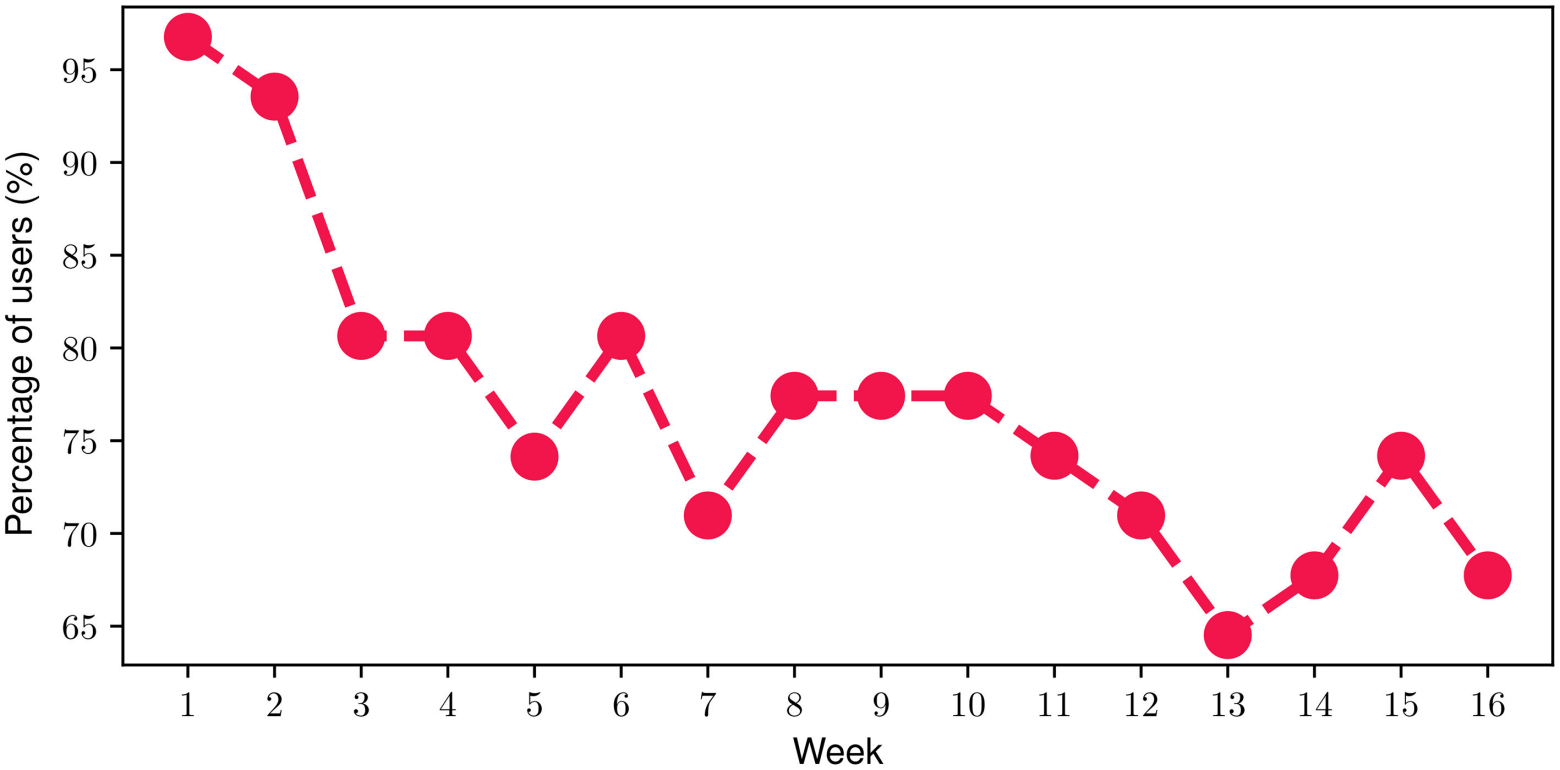
Percentage of participants engaging with the FES during each week of the FP.

With respect to the FES, we found out that on average, participants engaged with the FES for 74.5% of their time throughout the FP. In more detail, Figure 5 depicts the percentage of participants that engaged with the FES on a weekly basis throughout the FP. At the beginning of the FP (i.e., week 1), it can be noticed that almost all of the participants (more than 95%) engaged with the FES. Then, a slight decline can be observed during the next 2 weeks resulting in approximately 80% of the participants engaging with the FES by the end of Week 4. During the next 2 months of the FP (i.e., week 5-week 12), the proportion of participants attains an average value of 75.4% with a corresponding standard deviation of only 3.2%. The relatively high engagement ratio of 75.4% slightly drops to an average of 68.5% during the last month of the FP which can be attributed to a relatively reduced engagement level, as the study completion time point approaches. Overall, the mean weekly participant engagement with the FES was 76.8% throughout the study.

**Figure 6:**
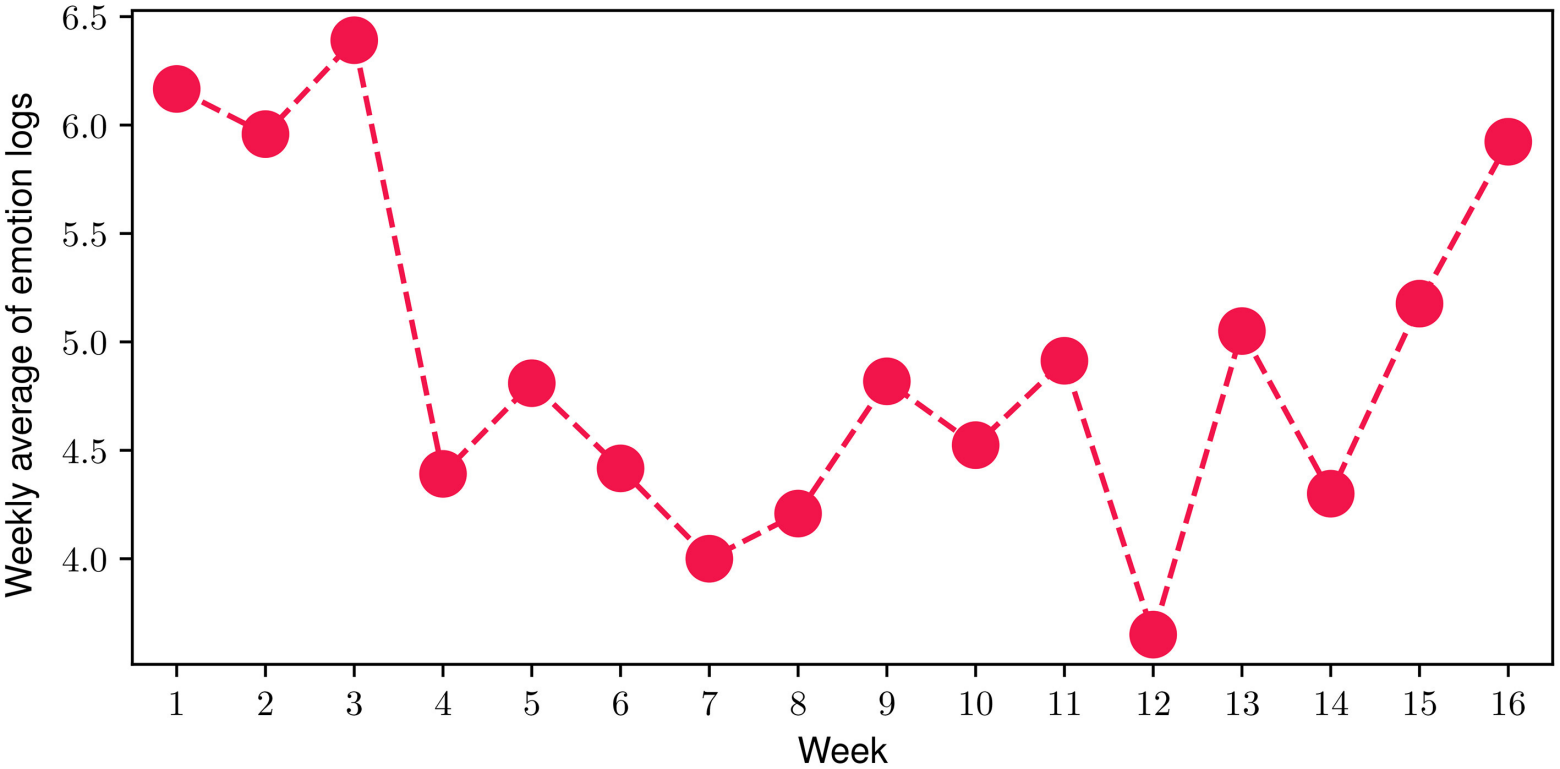
Weekly average of FES-triggered and manual emotion logs during the FP.

Regarding the emotion journaling activity throughout the program, we investigated the average number of total emotion logs (both FES-triggered and manually logged) registered by the participants. More specifically, in Figure 6 we present the number of total emotion logs for each of the 16 weeks of the FP, averaged over the number of the study participants during each week. The results indicate that during the FP, the average number of total emotion logs ranged from 3.65 to 6.4, with a mean value of 4.92 emotion logs per week, while 91.3% of them are also journalled (see Emotion Journaling in Section 2.3). In total, close to 2000 emotional moments have been logged during the 16 weeks of the study, with only 13.7% corresponding to days that participants did not engage with the FES.

### 3.4. Preliminary assessment of impact on mental health symptoms

**Figure 7:**
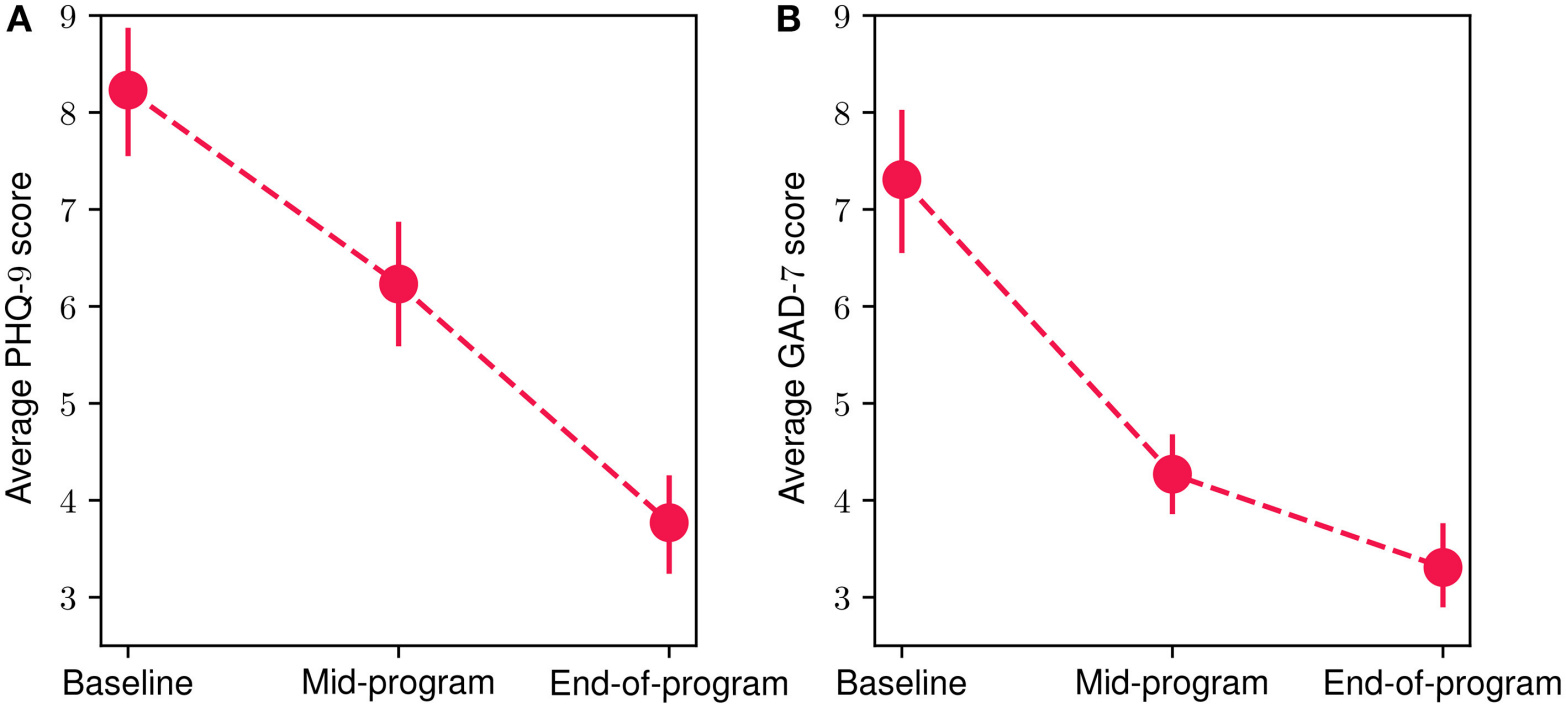
Mean participant PHQ-9 **(A)** and GAD-7 **(B)** scores at baseline, mid-program and end-of-program evaluations. The vertical bars represent the standard error.

Figure 7 showcases the mean scores from the mental-health-related questionnaires at baseline, mid-program (week 8) and end-of-program (week 16) evaluations. For both PHQ-9 (Figure 7A) and GAD-7 (Figure 7B) questionnaires, a decrease in mean participant scores can be observed, suggesting an important improvement in depressive and anxiety symptoms. More specifically, for the PHQ-9 assessment, the mean baseline score was 8.23 (*SD* = 3.44), while for the mid-program evaluation the obtained mean reduces to 6.23 (*SD* = 3.15), and reaches an average of 3.76 (*SD* = 2.48) at the end of the program. Similarly, for the GAD-7 questionnaire, at baseline the mean score was 7.30 (*SD* = 3.68), reducing to an average of 4.26 (*SD* = 2.08) at the mid-program assessment, before reaching a mean value of 3.3 (*SD* = 2.18) at the end of the program. These results suggest that the overall average mental health symptom reduction throughout the FP was 54.3 % for the depressive symptoms and 54.8% for the anxiety ones. The reduction was statistically significant in both cases (Wilcoxon signed-rank test with *p* < 10^−4^ and effect size *r* equals to 0.93 and 0.98 for the PHQ-9 and GAD-7 scores, respectively). More specifically, 87% of participants exhibited a reduction in depressive symptoms, while 83.8% had a reduction in anxiety symptoms. Additionally, 93.5% of participants presented a decrease in at least one the two symptom categories (i.e., PHQ-9 or GAD-7), while 77.4% of them showed a decrease in both of them. Referring to participants exhibiting clinically significant symptom improvement (62, 63), 51.6% of them showed improvement in depressive symptoms and 45% in anxiety symptoms. Finally, 74.2% of participants had improved by at least one severity level (e.g., changed from moderate to mild) in depressive symptoms and 71% in anxiety symptoms.

### 3.5. Preliminary assessment of impact on quality of life results

**Figure 8:**
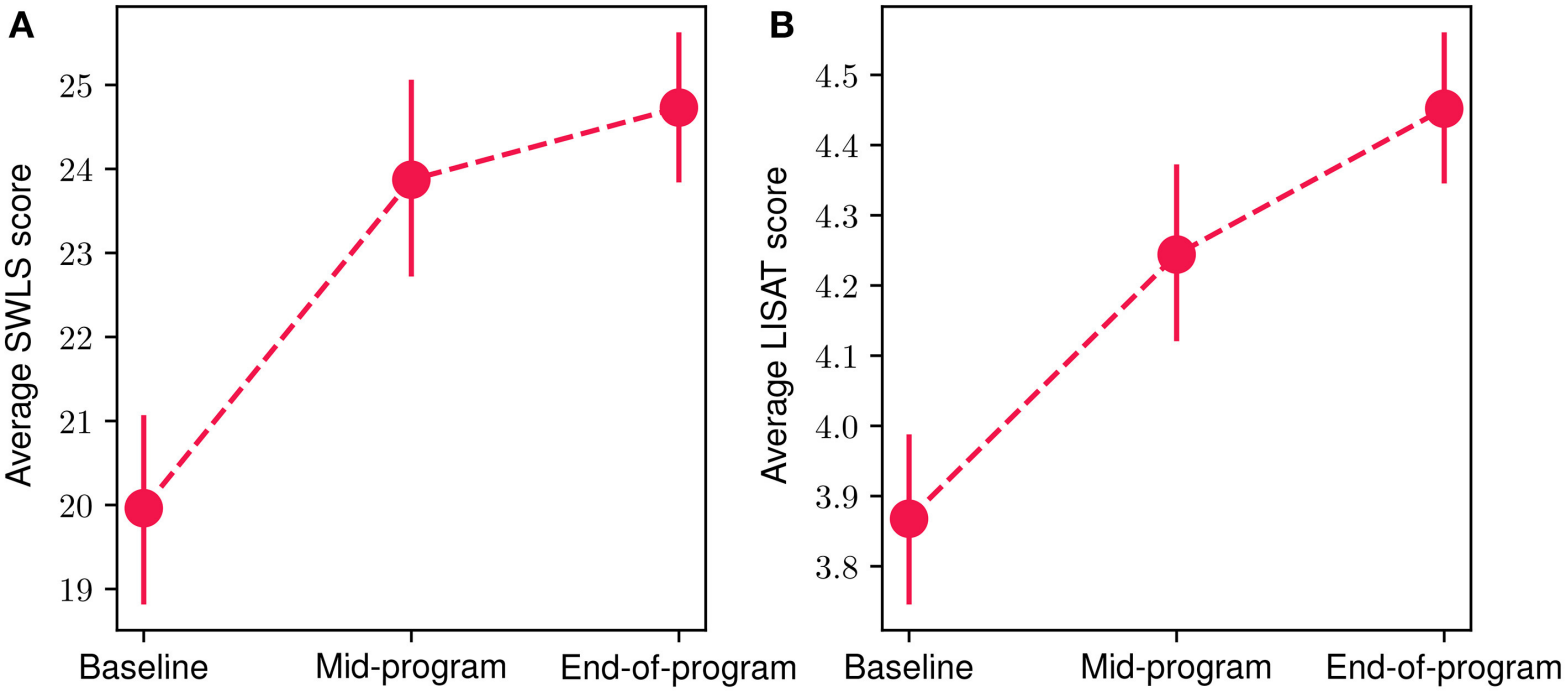
Mean participant SWLS **(A)** and LISAT-11 **(B)** scores at baseline, mid-program and end-of-program evaluations. The vertical bars represent the standard error.

In Figure 8, we present the mean scores for the SWLS (Figure 8A) and LISAT-11 (Figure 8B) questionnaires regarding quality of life aspects at the baseline, mid-program and end-of-program evaluations. An increase in both scores can be observed throughout the FP, with a 24% and 15% overall improvement for SWLS and LISAT-11 mean scores, accordingly. The reduction was statistically significant in both cases (Wilcoxon signed-rank test with *p* < 10^−4^ and effect size *r* equals 0.95 and 0.96 for the LISAT-11 and *SWLS* scores, respectively). More specifically, at the baseline evaluation, the mean SWLS score was 19.9 (*SD* = 5.42), at mid-program it increased to 23.8 (*SD* = 5.76) while at the end-of-program evaluation, it reached 24.7 (*SD* = 4.71). At the same time, the corresponding scores for the LISAT-11 questionnaire were at baseline 3.86 (*SD* = 0.59), at mid-program 4.24 (*SD* = 0.68) and at the end-of-program evaluation 4.45 (*SD* = 0.54). Finally, 80.7% of participants demonstrated an increase at the SWLS scores throughout the program, while 71.4% showed an increase at the LISAT-11 scores.

### 3.6. Preliminary assessment of participant self-assessment results

An additional tool used to assess the impact participants perceive that the FP has had, along with their accomplishments during the program, was the participant self-assessment questionnaire. Participant responses, following a Likert-type scale, are presented in Table 4. Overall, it is evident that participants anticipate that the program has had an important impact on them, as 100% of respondents (30 participants) state that the concerns that led them to the program have improved during their participation. Additionally, almost 97% of participants feel that they have made progress toward the goal they had set at the beginning of the program, while the same percentage expressed that their everyday lives have improved. Finally, all participants responded that they learnt to think more clearly to reduce distressing emotions/behaviors and more than 85% of them increased their ability to recognize, name, and/or appropriately express their emotions.

**Table 4:** Participant responses to the self-assessment questionnaire.

**Survey questions**	**Participant responses**, *n* **(%)**
My concerns that brought me to the program have improved as a result of the services provided.	Strongly agree: 17 (56.7%) Agree: 13 (43.3%) Neither disagree nor agree: 0 (-) Disagree: 0 (-) Strongly disagree: 0 (-)
I feel I made progress toward my set goal.	Strongly agree: 18 (60%) Agree: 11 (36.7%) Neither disagree nor agree: 1 (3.3%) Disagree: 0 (-) Strongly disagree: 0 (-)
My everyday life has improved.	Strongly agree: 15 (50%) Agree: 14 (46.7%) Neither disagree nor agree: 1 (3.3%) Disagree: 0 (-) Strongly disagree: 0 (-)
I learned to think more clearly/accurately to reduce distressing emotions or behaviors.	Strongly agree: 22 (73.3%) Agree: 8 (26.7%) Neither disagree nor agree: 0 (-) Disagree: 0 (-) Strongly disagree: 0 (-)
I increased my ability to recognize, name, and/or appropriately express my emotions.	Strongly agree: 19 (63.3%) Agree: 7 (23.4%) Neither disagree nor agree: 3 (10%) Disagree: 1 (3.3%) Strongly disagree: 0 (-)

## 4. Discussion

### 4.1. Key findings

In this study, we have presented the results of a RWD feasibility study that serves as a PoC for a digital data-driven mental health program (i.e., the Feel Program) for people suffering from MDD and/or GAD. In this context, an experimental protocol involving the deployment of the 16-week Feel Program at a population with mild or moderate MDD/GAD, has been designed and executed. Our first aim was to explore the feasibility of such a program, as captured by the responses of potential participants' to the recruitment campaign. We, therefore, implemented a relatively broad campaign, with the aim of exploring the various personas, mental health conditions and demographic profiles of individuals that were more interested in the program and, therefore, in joining the study. At the same time, the study protocol did include a set of strict inclusion and exclusion criteria (e.g., exclusion of specific mental health disorders, no psychotropic medication, etc.), ensuring that only participant profiles aligned with the study scope were finally enrolled. Therefore, the recruitment campaign attracted a high number of respondents to the demographics and eligibility questionnaire, indicating that there is a great need for mental health support resources for numerous conditions. The combination of the high number of responses to the broad recruitment campaign and the presented exclusion and inclusion criteria led to an eligibility ratio of 8.5%. In particular, a high prevalence of the exclusion disorders (i.e., personality disorders, psychotic disorders, bipolar disorder, eating disorders) was observed, with 3 out of 4 applicants reporting the existence of at least one of the above. Additionally, we noticed that the vast majority of respondents were females (76.2%), frequently low-to-mid income level (44% with less than 15, 000 annual income) and often with a basic level of education (32% high-school graduates or no schooling completed). This is in line with findings from previous studies (18, 65) that report that income and education levels can limit access to mental health services. Finally, it should be highlighted that 1 out of 3 respondents reported moderately severe or severe depressive symptoms and 1 out 4 severe anxiety symptoms. The gender distribution of the eligible participants that joined the study was more balanced, with 62.5% female participants and 37.5% males, and the 31−40 age group has the highest proportion of participants (41.7%). Overall, 70% of participants were aged 40 years or younger, while interestingly 12.5% were older than 50, suggesting that technology-driven programs could be attractive to older adults too. Finally, the distribution of baseline symptom severity was well balanced with regards to MDD with 50 and 40% of participants exhibiting mild and moderate depressive symptoms at the beginning of the study respectively, and 50 and 30% reporting mild and moderate anxiety symptoms, respectively.

The second aim of the present study, after its feasibility was established, was to explore the main hypothesis which revolved around the fact that a remote, data-driven and personalized program would boost the participant engagement, while a significant improvement of their depressive and anxiety symptoms would be observed. In order to support our hypothesis, our analysis focuses on three main sections: (i) in-the-wild Feel technology validation; (ii) acceptability and participant engagement and (iii) preliminary assessment of impact on depressive and anxiety symptoms, as well as on quality of life aspects. In the following, we discuss in more detail the key findings of our investigation with regards to these aspects. In the context of technology validation, we aimed to demonstrate that the enabler of the data-driven nature of the FP, the Feel Emotion Detection technology, can be deployed in real-world settings maintaining similar performance levels compared to our testing environments. Successful deployment of technology is dependent on; (i) system-design-specific factors impacting performance (e.g., high precision and minimal bias toward specific emotions or individuals), and (ii) successfully withstanding challenges associated with real-world applications, such as unknown emotional stimuli, noise signal artifacts and stochastic participant behavior. The results of this study show a very high average precision level (87%) in identifying emotional events for this group of individuals suffering from MDD and/or GAD. Moreover, positive and negative emotional events were detected in a balanced (54 and 46% respectively), suggesting that there was a minimal bias toward either valence category. Finally, we have shown that, for the majority of the participants (> 70%), their FES-triggered events were registered on average as high-intensity (i.e., with a participant-perceived intensity level ≥6). These findings indicate that the Feel Emotion Detection technology performs well enough to capture significant emotional events from any participant.

The next step toward validating our initial hypothesis includes assessing the acceptability of and participant engagement with the FP. Regarding the former, participant responses to the user feedback survey collected after the completion of the 16-week study suggest that participant satisfaction levels were considerably high with over 65% (20 participants) reporting very or extremely high satisfaction levels. Additionally, 4 out of 5 participants were at least very likely to recommend the program to someone. These findings imply that the FP sufficiently addressed their needs and met their expectations. Furthermore, the data-driven nature of the program—in the form of the data-driven weekly sessions with the provider and the FES—was recognized in the survey responses as one of the most important features of the program. Specifically, more than 90% of participants identified the weekly data-driven sessions as the most important feature of the program, while 70% selected the FES. Participants also reported particularly liking the data integration to the program and the in-the-moment interventions, powered by the FES-triggered emotion logs.

Regarding participant engagement, we focused on participant retention levels throughout the program, as well as the degree of engagement with the different components. An overall 65% retention rate was observed with most discontinuations from the program occurring during its 1st month while no participants dropped out after the midpoint of the program. This could be related to the initial introductory period required for participants to acquaint themselves with the various program components, and the level of commitment, effort, and time resources required. Equally, this could possibly be attributed to the structure of the FP and its use of the theoretical framework of the Stages of Change (i.e., Contemplation, Preparation, Action) (66). During the first introductory session, the participant sets their program goals and then when they are faced with taking action, they either regress into Contemplation stage and drop out of the program or progress into Action stage and increase their engagement in the program.

Turning our attention toward participant engagement, a wide range of extracted metrics referring to all program components indicate high engagement levels. Overall, completed participants spent on average 60 min per week in the Feel app, accessing it on average 2.5 times per day. This equated to an interaction with at least one of the FP components on average for approximately 3.1 days per week. Additionally, the term weekly Mental Health Actions was introduced as a metric to capture participants' weekly activity during the week which reached an average of 13 actions per week for the completed ones. The highest amount of activity was the on average 5 mental health exercises accessed *via* the mental health resource center per week, followed by the emotion journaling feature, with completed participants registering an average of 4.92 emotional events, either FES-triggered or manually input, per week. Finally, a very high rate of attendance to the weekly sessions with the provider (96.9%) was also observed.

With respect to the main data-driver in the FP, the FES, completed participants engaged with it for 74.5% of the time while they were on the program, with an average of 76.8% of them using the sensor on a weekly basis. An interesting observation was that increased engagement with the FES boosted overall completed participant engagement in terms of both increased FES-triggered emotion logs, and also increased manually registered emotional events. The former seems quite intuitive, as the more physiological data are available, the more events can be detected. However, what is particularly interesting is that increased FES engagement was also highly associated with a higher number of the manual logs. More specifically, we observed that on the days that completed participants engaged with the FES, the number of manual emotion logs more than doubled, compared to the days without any FES engagement. The contribution of the FES to emotion journaling can be quantified by an approximate 5.5-fold increase in the probability of an emotion log occurring on days with FES engagement. The FES is considered a driving factor for overall participant engagement with the program, since it is strongly associated with the majority of the emotion logs, which are in turn linked with the various other components of the FP (e.g., data-driven weekly sessions, app usage, weekly exercises, etc.).

Having discussed the in-the-wild performance of the technology and the fact that the data-driven nature of the program significantly enhances engagement, we lastly focused on depressive and anxiety symptoms, as well as on quality of life levels. For both MDD and GAD symptoms—reflected by PHQ-9 and GAD-7 scores, respectively—a continuing decrease in average scores was observed from baseline to mid-program, and all the way to the end-of-program evaluations, with 9 out of 10 participants reporting symptom reduction for at least one symptom category. Overall, the average reduction in both scales during the 16-week program was over 50%. For almost 3 out of 4 participants the severity of their symptoms decreased by at least one severity level, while a clinically significant improvement was characterized for almost half of the participants. This symptom improvement was followed by a subsequent increase of 24% on average in quality of life metrics. It should be noted that this study did not include participants with severe depression or anxiety symptoms, in order to exclude the effect of pharmacotherapy on symptom improvement. We anticipate that the inclusion of more severe cases will significantly enhance the total average reduction of mental health symptoms in a 16-week program like FP (67).

In summary, the study results support that very high levels of participant engagement with a 16-week personalized data-driven digital mental health program, with its data-driven nature—coming from the use of a wearable sensor—being a key engagement factor. Moreover, the increased participant engagement levels and the data enhancement of the provided mental health support, may be linked to a significant reduction of depressive and anxiety symptoms, as well as an improvement in quality of life. Table 5 summarizes the highlights of this study.

**Table 5:** Highlights of the study.

**Key outcomes**	
**Engagement metrics**	
Users active during the week	96%
Users engaging with the FES during the week	76.8%
Weekly time in app	60 min
Weekly mental health actions	13
Weekly mental health exercises	5
Weekly emotion logs	4.92
Average notification precision	87%
Session with the provider compliance	96.9%
**Impact on mental health outcomes**	
Average improvement of depressive/anxiety symptoms	54.3%/54.8%
Participants with clinically significant depressive/anxiety symptom improvement	51.6%/45%
Participants with improvement in at least one the two symptom categories	93.5%
Participants with improvement in both symptom categories	77.4%
Participants that improved by at least one severity level in depressive/anxiety symptoms	74.2%/71%

### 4.2. Further observations

**Figure 9:**
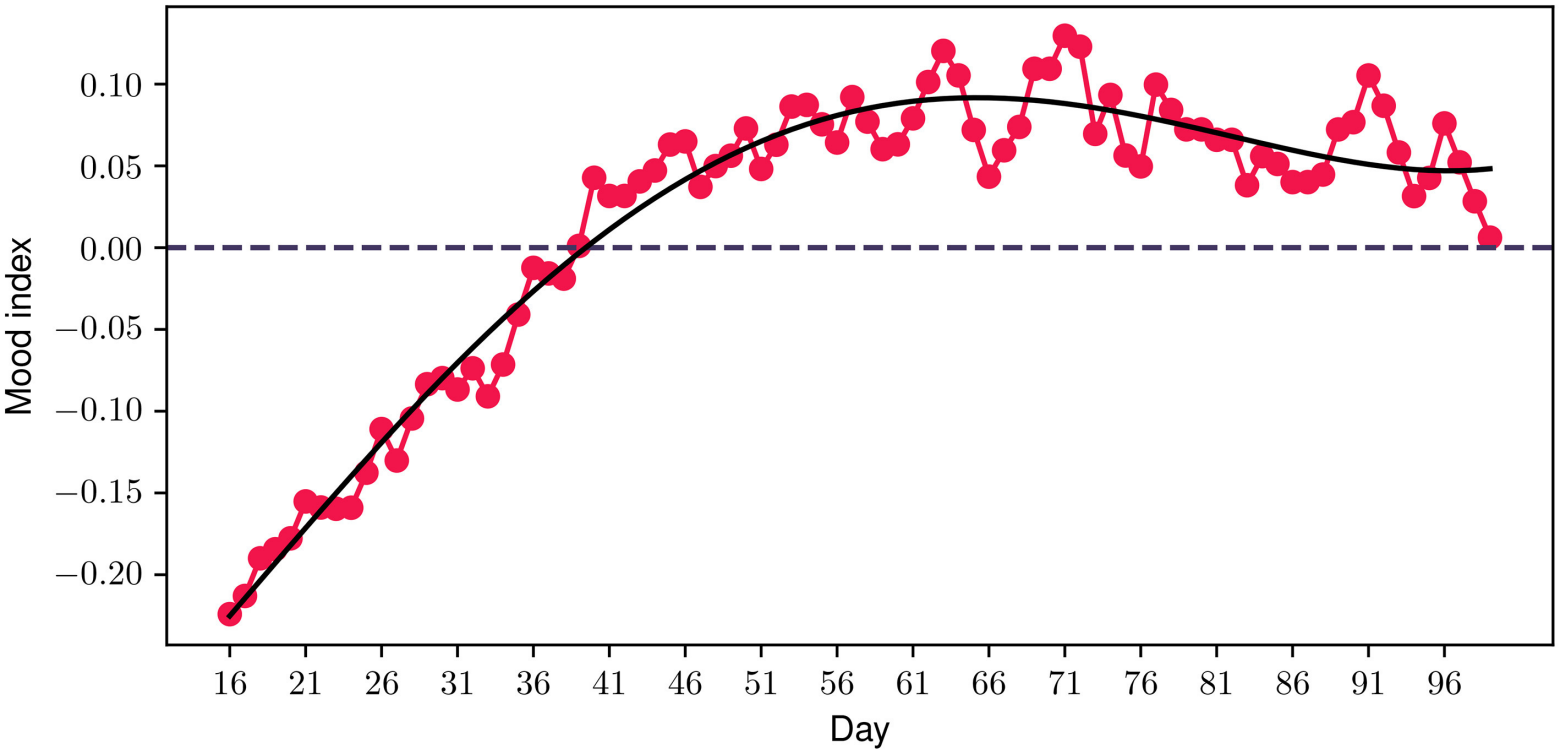
Participant mood index progression during the program. The solid black line represents a polynomial fit.

Having discussed our initial hypothesis which evolved around the feasibility, acceptability, engagement and potential impact on mental health symptoms of the FP, a few interesting observations are presented. Considering that the emotion journaling aspect constitutes a core component of the program, we introduced an additional metric—the mood index—to capture the progression of participants' emotional patterns throughout the program. This new metric is derived from a combination of the different positive and negative emotions logged by the participants, where each emotion valence category is mapped accordingly to a positive (+1) or negative (−1) value, accordingly. The daily value is then derived from the average value of all emotions logged during each particular day. Figure 9 illustrates the 30-day centered rolling average of the mood index for the duration of the study. A very interesting observation is that the average values of the mood index reach very low negative values when participants join the study, indicating that the vast majority of emotions experienced at the start of the study are negative. However, a steadily increasing trend is observed from the beginning of the program, which aligns with the above-mentioned improvement in depressive and anxiety symptoms and quality of life. Close to the 40 day time point, the index transitions into a positive value, where it stabilizes for the rest of the program.

**Figure 10:**
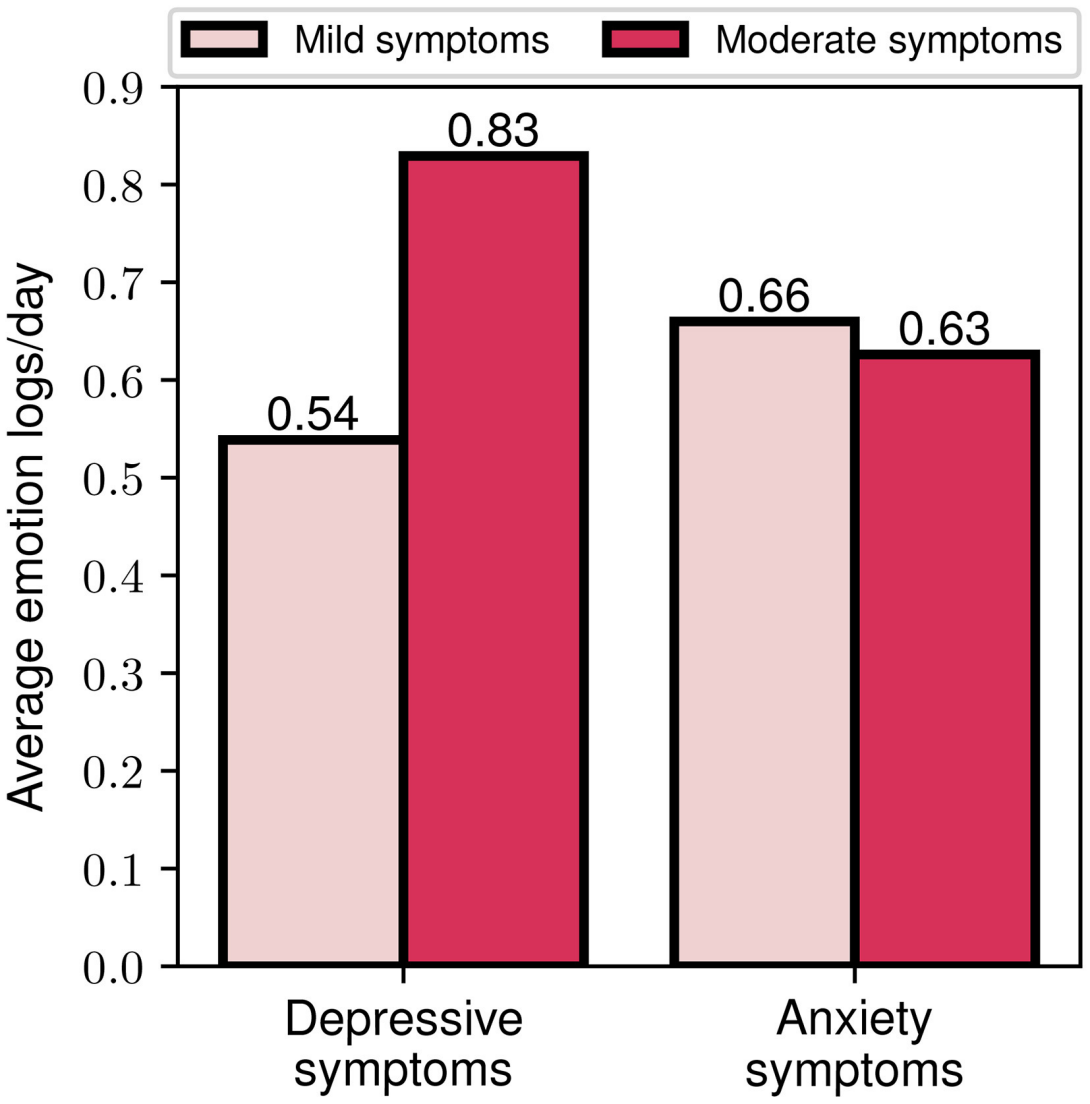
Average emotion logs per day for mild and moderate depressive and anxiety symptoms baseline assessment.

As an additional step toward exploring the importance of the data-driven component of the FP, we explored the level of engagement of different participant cohorts based on their baseline symptom severity. In this context, the average number of emotion logs per day was selected as a measure of engagement, as it constitutes one of the most important engagement metrics. In Figure 10, the average engagement levels throughout the study for participants with mild and moderate symptom severity at baseline are presented. We should highlight that for the depressive symptoms cohort, a clear difference between the two groups can be noticed, with the moderate severity group exhibiting a more than 50% higher engagement compared to the mild severity (0.83 vs. 0.54). When grouping participants according to their anxiety symptom severity however, no significant differences in engagement between the two groups were observed (0.63 vs. 0.66). These observations may serve as an indicator of greater participant engagement when symptom severity is higher, especially for those with depressive symptoms.

**Figure 11:**
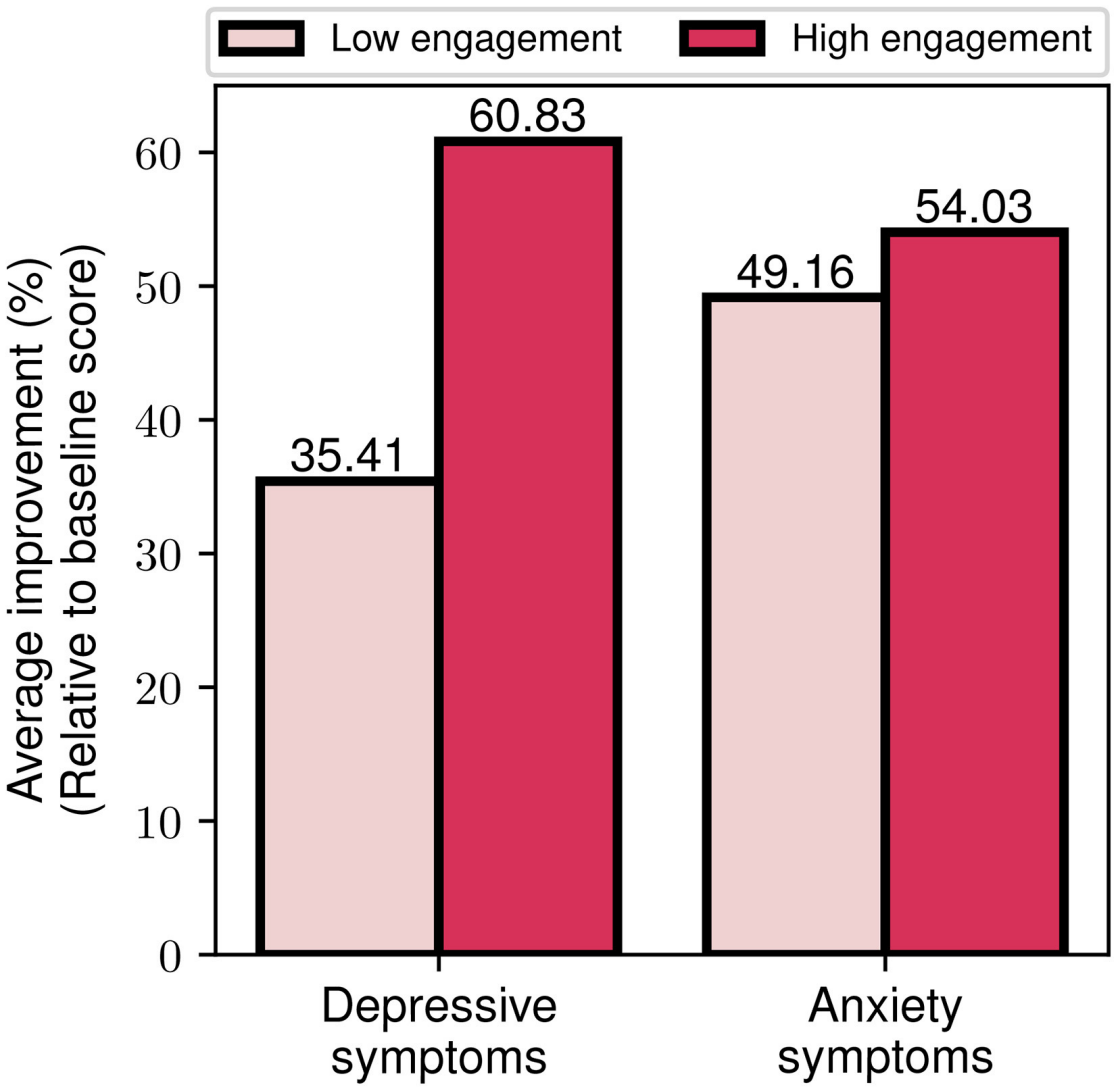
Average depressive and anxiety symptom improvement for low and high engagement participant groups.

Finally, we explored the effects of differing engagement levels (i.e., low and high engagement) on the program's impact on symptom severity. By utilizing the aforementioned index of engagement, we assigned participants to the high (or low) engagement group, if they had more (or less) than 1 emotion log every two days. This threshold was selected based on the average weekly rate of emotion logs for all participants. In Figure 11, we present the percentage reduction in depression and anxiety symptom severity for the two engagement cohorts. It is notable that the high engagement cohort shows a more than 70% greater improvement in depressive symptoms compared to the low engagement cohort (60.8 vs. 35.4%). The outcomes are more balanced for the case of anxiety symptoms, where the high engagement cohort presents a 10% higher improvement compared to the low engagement (54 vs. 49.2%). Altogether, these findings serve as an indication that increased engagement with the program, as reflected by participants' emotion logging activity, seems to be associated with greater improvement of depressive and anxiety symptoms.

### 4.3. Limitations

When interpreting the results presented in such a paper, study limitations should always be considered. The most important among these is the lack of a control group, which could provide further evidence as to whether the overall symptom and quality of life improvement can be attributed to the intervention or not. Secondly, the sample size of the study was relatively small with 31 participants actually completing the program. Additionally, the different cohorts include a small number of participants, so the respective insights should be treated with caution. Thirdly, a more diverse demographic group (e.g., expanded age groups, balanced gender groups, racially diverse groups, etc.) of participants could be included in future studies, in order to generalize the findings to a general demographic audience. Additionally, the study includes only participants with mild and moderate depressive and anxiety symptoms severity. Examining the impact of the program on severe cases in a follow-up study would be valuable. Finally, the level of mobile device proficiency was not monitored and its impact on participants' engagement was not assessed.

### 4.4. Conclusions

In summary, this study aimed to explore the hypothesis that a digital, biodata-driven mental health program, the Feel Program, would introduce multiple benefits for people suffering from mild or moderate MDD and/or GAD. Therefore, we first focused on providing evidence supporting the feasibility of this program. Feedback from the participants revealed very high satisfaction scores across the different components of the program, and highlighted the significance of their data-driven nature. Moreover, overall completed participant engagement remained at high levels throughout the program and was significantly boosted by the use of the sensor. Additionally, we have shown that the key differentiating program component—the Feel Emotion Sensor and the Feel Emotion Detection—can be successfully deployed in-the-wild and can accurately detect significant emotional events. Overall, participants exhibited significant improvement on depressive and anxiety symptoms, with almost all of them showing symptom reduction at the end of the study. However, a controlled trial will be required to demonstrate further evidence of the clinical efficacy of the program. Finally, our indicators regarding the value of using personalized data - mainly collected by the FES—suggest enhanced engagement for groups with more severe depressive symptoms, as well as greater symptom improvement in participants with higher engagement throughout the program.

## Data availability statement

The original contributions presented in the study are included in the article/supplementary material, further inquiries can be directed to the corresponding authors.

## Ethics statement

The studies involving human participants were reviewed and approved by the Ethics Committee of the National Kapodistrian University of Athens, First Department of Psychiatry, Eginition Hospital. The patients/participants provided their written informed consent to participate in this study.

## Author contributions

All authors listed have made a substantial, direct, and intellectual contribution to the work and approved it for publication.

## Conflict of interest

Authors CT and GE are employed by Feel Therapeutics Inc., receive a salary and own a large share of the company stocks. DA and PF are employed by Feel Therapeutics Inc., receive a salary and own options of the company. JA is an advisor to and holds options of the company. The remaining authors declare that the research was conducted in the absence of any commercial or financial relationships that could be construed as a potential conflict of interest.

## 5. Publisher's Note

All claims expressed in this article are solely those of the authors and do not necessarily represent those of their affiliated organizations, or those of the publisher, the editors and the reviewers. Any product that may be evaluated in this article, or claim that may be made by its manufacturer, is not guaranteed or endorsed by the publisher.

## Footnotes

^1^myStrength, AbleTo, Talkspace, Lyra, ginger, Meru Health.

^2^myFeel.
